# The effect of a knee brace in dynamic motion—An instrumented gait analysis

**DOI:** 10.1371/journal.pone.0238722

**Published:** 2020-09-10

**Authors:** Hannah Lena Siebers, Jörg Eschweiler, Jan Pinz, Markus Tingart, Björn Rath

**Affiliations:** 1 Department of Orthopedic Surgery, University Hospital RWTH Aachen, Aachen, Germany; 2 Department of Orthopedic Surgery, Klinikum Wels-Grieskirchen, Wels, Austria; Texas State University, UNITED STATES

## Abstract

**Background:**

Osteoarthritis (OA) is a common problem in the older population. To reduce pain and stress in the affected knee joint compartment, a functional knee brace is often prescribed by physicians to protect it from high loads.

**Objectives:**

An instrumented gait analysis should evaluate how the 4-point knee orthosis for varus or valgus load relief (M.4s OA) changes the kinematics of the knee, especially in the frontal plane.

**Methods:**

17 healthy participants took part and were analyzed with an inertial sensor system (MyoMotion) giving continuous, objective information on the anatomical angles. The measurements were made both without wearing a knee brace and with the brace in different settings.

**Results:**

The results show a significant reduction in the maximum knee abduction and raised knee adduction. The knee brace, with a strong adjustment in varus or valgus orientation, caused a shift of maximum ab-/adduction in the proposed direction in 69% and 75% of the dynamic tests, respectively. The knee motion in the frontal plane shows individual movement patterns.

**Conclusion:**

The use of the brace leads to significant changes in the knee’s movement. Patient-specific movement patterns may explain different effects of functional knee braces on individual persons. Inertial sensors have been shown to be a low-cost, easy-to-use option for individual movement analysis and further personalized therapy.

## Introduction

Osteoarthritis (OA) is the leading cause of musculoskeletal disability in most developed countries [1–4]. The prevalence increases with age and is higher in women than in men [4]. OA is characterized by a loss of cartilage in the affected joint, with the knee being the most frequently affected of all joints [1–4]. The main problems of OA-patients are pain and reduced mobility [1], therefore, patients need walking frames and analgesics [5]. The burden of OA on the population, healthcare system, and the overall economy will continue to increase in the future. From a biomechanical point of view, increased joint loads can cause OA through means of increased joint loads which in turn leads to the destruction of the synovium, bone, and cartilage [5]. Catabolic cytokine cascades and the production of inflammatory mediators also play a significant role [3].

Some patients of OA do not wish to undergo surgery, accordingly, it has been analyzed that after consultations with an adequate specialist and a biomechanical analysis, an effective, non-invasive treatment plan can be created [5,6]. Corrective devices, like braces or insoles, along with strength and exercise training has been analyzed to be beneficial in conservative OA treatment [2,5,7,8]. In all cases of conservative treatment, the goal is to decrease the load and forces on the affected knee compartment. Most simply the loads are decreased by weight loss, active by strength and exercise training to increase muscle strength, and/ or passive by external forces of corrective devices to change the leg axis [5]. The argument for conservative management of knee OA has strengthened as the last mentioned methods have shown to be helpful in reducing knee joint instability, which is a commonly described symptom of knee OA and is correlated with a decreased ability for patients to perform activities of daily living [9]. When prescribed by a physician, a knee brace can play a prominent role in the non-operative management of knee injuries by reducing pain and stress in the affected knee joint compartment. The brace achieves this by providing adequate support, protecting the affected area from high loads, and reducing medial or lateral knee joint loading [7]. This load shift can be attained by designing the knee brace to apply precise, counteracting valgus or varus forces on distinct components of the knee joint complex. An analysis of the effectiveness of knee braces in conservative knee OA management produced results suggesting that not only can knee braces be an effective means for relieving pain, but it can also decrease joint stiffness and the necessity for pain medication with minimal adverse effects [5]. According to Lee et al, knee braces have even demonstrated to be a cost-effective method to bridge and delay surgery [10]. Chronic use of knee braces for OA treatment was found to be successful, with the most failures occurs early during the first 6 months [11]. As potential complications of brace treatment leg swelling, venous thrombosis, and thromboembolism were described [11].

**Fig 1:**
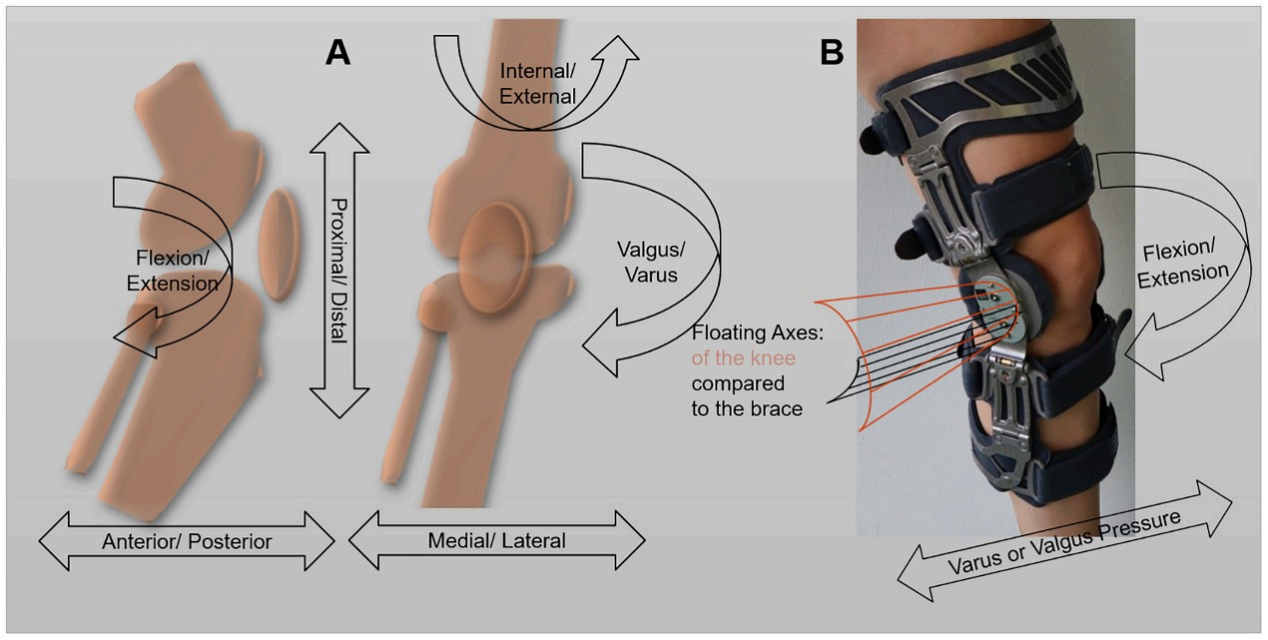
Knee kinematics. (A) Knee kinematics from lateral and frontal view in comparison to (B) brace kinematic of the 4-point knee orthosis for varus or valgus pressure relief and stabilization (M.4s OA, medi GmbH & Co. KG, Bayreuth, Germany).

Knee kinematics is complex (Fig 1A). It includes six degrees of freedom like flexion/ extension, ad-/ abduction, and internal/ external rotation. The knee flexion and extension axis is a floating one [12].

In contrast to the knee, brace kinematics are less complex as it does not include a real floating axis (Fig 1B). Braces consist of two frames that are connected via an artificial joint e.g. a polycentric hinge. They are fixated by fastening straps around the thigh and shank. The artificial joint causes the tibial frame to perform a translational movement relative to the femoral frame [13].

Previous studies have pointed out that further dynamic investigation is necessary to prove the effectiveness of knee braces for OA treatment. In most cases, former investigations on this issue have only examined the immediate effect of valgus knee braces on aspects such as knee loading [14] and adduction moment [1,15], muscle co-contraction [1,16], functional joint space and joint contact locations [17], pain [2,18], and balance rather than the lasting changes after a treatment period [1,9]. Literature research has shown that an analysis of the effect of varus knee braces was less common than valgus [7]. A detailed outstanding overview of the current scientific progress and standing can be found in e.g. [2,7,8,15,19]. Studies on knee kinematics have allowed for in-depth analysis of restrictions in movement caused by the brace, improving comprehension of secondary problems, and overall wearability. Furthermore, our analysis of the kinematic effects on knee braces, when combined with previously presented studies on knee loading and muscle activation, presents a more complete view of the effect of knee braces.

Inertial Measurement Units (IMUs) are a low-cost and easy-to-use alternative method to complex static optoelectronic measurement systems for determining kinematic data [20]. They consist of 3 orthogonal accelerometers, gyroscopes, and a magnetometer (or parts of it) [20]. Wearable IMUs can be used in a broad range of circumstances ranging from a laboratory environment to a sports field, and as they become more popular [21,22], it is important to continue to analyze them and further our understanding. Therefore, we aim to demonstrate the effectiveness of IMUs for the analysis of kinematic knee brace effects.

In our study, the effects of a knee brace for the treatment of unicompartmental OA were investigated using an IMU approach, focusing on the brace’s influence on knee kinematics, especially in the frontal plane. Along with differences in kinematics, different varus and valgus adjustments and slippage of the knee brace can cause misalignment of the knee and brace rotation axes, which might influence the global knee motion. Our focus was on the frontal plane because this is where the ab-/ adduction manipulation took place and where the knee joint position change occurred. We hypothesized that the pressure relief provided by the varus and valgus knee braces causes adapted movement patterns like increased adduction in addition to movement restriction displayed by decreased knee flexion.

## Materials and methods

### Test setup

A test setup was designed to study the influence a brace intended to reduce the load in the medial or lateral compartment of the knee joint has on the knee kinematics using an IMU system. There were 17 healthy participants with normal knee alignment included in this study; 13 men, 4 women, mean age of 28 ± 4 years. The mean weight was 77 ± 11 kg and the mean height was 180 ± 11 cm, resulting in a mean body mass index (BMI) of 24 ± 2 kg/ m^2^. Subjects with knee pain or problems or any other musculoskeletal or neurological diseases which could influence gait and posture were excluded. Healthy participants were chosen because in those cases the knee joint itself did not have any variations related to OA deformities ensuring our results would not be influenced by OA related parameters or effects. Without restrictions like pain, we were able to analyze the brace’s effect on the knee kinematics during activities like walking. Moreover, we were able to better compare our observations to the physiological knee function because testing on healthy patients allowed us to measure the effects of valgus and varus bracing on the same knee.

We used the 4-point knee orthosis M.4s (M.4s OA, medi GmbH & Co. KG, Bayreuth, Germany) for varus or valgus pressure relief and stabilization (Fig 1B). The M.4s is a functional brace, which could manipulate the knee orientation in the case of OA.

The tibiofemoral kinematic for both legs was acquired using MyoMotion, an IMU-system developed by Noraxon (Noraxon U.S.A. Inc., Scottsdale, USA). The MyoMotion uses advanced, medical-grade IMUs that measure 3D motion with an accuracy level within 1 to 2 degrees of legacy camera-based systems [23–25]. They allow for a level of flexibility not possible in a lab-based system [24]. Seven sensors were positioned based on anatomical landmarks: one sensor on each foot, shank, and thigh and one sensor central at the pelvis [26]. The sensors are fixed at one-third the length of the thigh above the brace and one-third the length of the shank under the brace. Based on the sensor data, the orientation angles (yaw (x), pitch (y), and roll (z)) of body segments (thighs, shanks, feet, and pelvis) and the anatomical angles of the ankle, knee, and hip were calculated. Anatomic angles are calculated based on the neutral zero method. Even in the case of small differences in knee alignment, all measurements were referenced to the individual initial knee position. All measurements were done with a sampling frequency of 100 Hz [24].

### Dynamic testing

Testing began with the calibration of the IMU-system by having the participant stand with their knees fully extended in a fixed and neutral position. Then, in the first step of dynamic testing, all subjects were instructed to walk 25 m at a self-selected, comfortable speed without a brace over a flat surface while the IMU-system collected kinematic data. Participants were advised to adopt a comfortable walking velocity instead of a default speed to minimize the amount of external influences and boundary conditions. In this way, the later addition of the brace would be the only difference between experiments. Dynamic testing with the brace was conducted in a similar manner. First, the brace was strapped in accordance with the manufacturer’s instructions. Then, the complete “system” (knee with a brace) was checked to ensure that both the femur and the tibia were correctly supported by the knee brace. Next, five different alterations of the brace were tested: one neutral, and a light or strong varus or valgus adjustment. The starting point for each adjustment was the neutral orientation, where no load should be applied to the knee. The light and strong adjustments were defined as a quarter and full rotation of the screw for pressure relief on the varus or valgus brace. The order of measurements was randomized and the brace was always worn on the right side. As a result of complete randomization, the numbers of participants with the same order of measurement conditions were unbalanced and in addition to their small number, no order effect was analyzed. All measurements were made individually for each participant by the same investigator, trained in using the MyoMotion system.

### Data analysis

**Fig 2:**
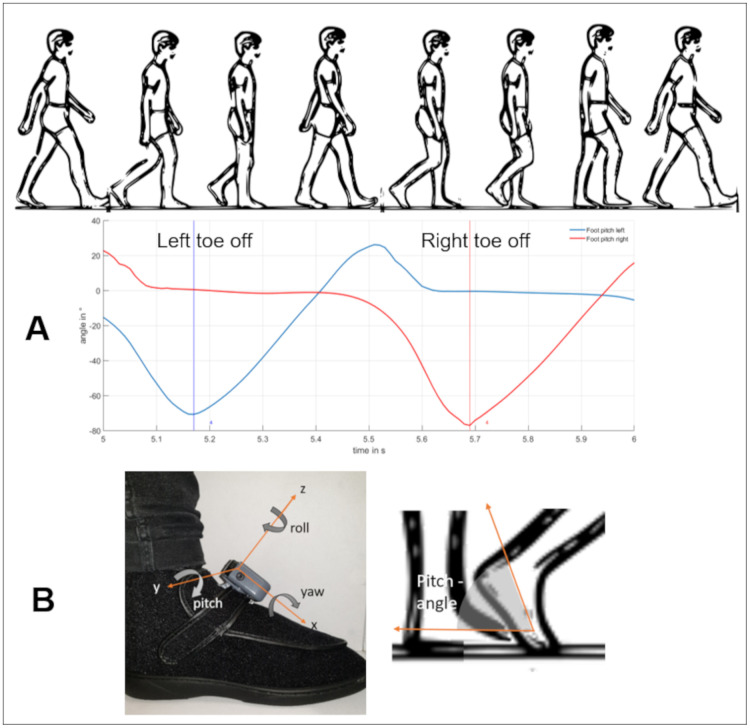
Determination of the terminal contact, based on the maximum deflection of the pitch angle. (A) The gait cycle and the measured pitch angle of the feet. (B) The definition of the orientation axis and the pitch angle of the foot sensor.

Measurement data was systematically processed and analyzed using calculations made with self-written Matlab-scripts (MatWorks R2019b, MathWorks, Natick, MA, USA). In order to analyze the brace’s effect, it was critical to measure the knee’s kinematics, especially in the frontal plane (ab-/ adduction plane). First, the angle data (anatomical and orientation angles) from all subjects was divided into single strides. Each stride began with the initial foot contact, followed by the stance phase, the toe-off, the swing phase, and ended with the following initial contact [27]. Based on the kinematic data, minima of the orientation angle curve of the feet in the sagittal plane (pitch, y-axis) were established as times for terminal contacts or toe-off events [28,29] (Fig 2). Next, the time axis was normalized to percent of the gait cycle. Using a Matlab function (interp1), each stride was interpolated to 100 properties. To shift the data ensuring an initial contact beginning of the gait cycle, the stance and swing phases we assumed to be 60% and 40%, respectively, of the complete gait cycle.

To eliminate the influences of start and stop events, the first and last stride of each measurement was excluded. Since each subject took a different number of strides over the 25 m, 15 strides of each leg, were taken for analysis. Based on the mean angle curve of 15 strides over one gait cycle, the extrema were calculated for statistical analysis. Besides the statistical evaluation, these curves were also visually inspected and analyzed, in comparison with the angle course presented in previous literature and between the different participants.

Based on previous research reports, an appropriate sample size was determined [14,16,17,19]. Statistical analyses were performed using the software IBM^®^ SPSS Statistics (IBM^®^ SPSS Statistics v. 25, IBM Cooperation). The normal distribution of the dependent variables was examined using the Shapiro-Wilks test and was not confirmed for most variables. Therefore, nonparametric tests were used. The effects of wearing a brace were evaluated by comparing results between measurements taken in a neutral adjustment, with and without the brace, using a Wilcoxon test. The effect size r is equal to $r=\frac{z}{\sqrt{N}}$ (with z-value from the associated Wilcoxon test and N as the number of pairs). It is presented as the coefficient of determination r², explaining the collective variance of the analyzed variables. To compare between the different conditions of the brace, a Friedman test was calculated with pairwise Post Hoc analysis and the brace adjustments as the independent variables. There were three groups: neutral, light, and strong brace adjustment. The pairwise comparison was not made in case of a negative hypotheses test. Statistically significant differences were set at p<0.05.

This research was approved by the Ethics Committee of the University Hospital RWTH Aachen and written informed consent was obtained. The research related to human use complies with all the relevant national regulations, institutional policies and was performed in accordance with the tenets of the Helsinki Declaration, and has been approved by the authors’ institutional review board (EK 251/18).

## Results

### Physiological knee movement in 3 dimensions

**Fig 3:**
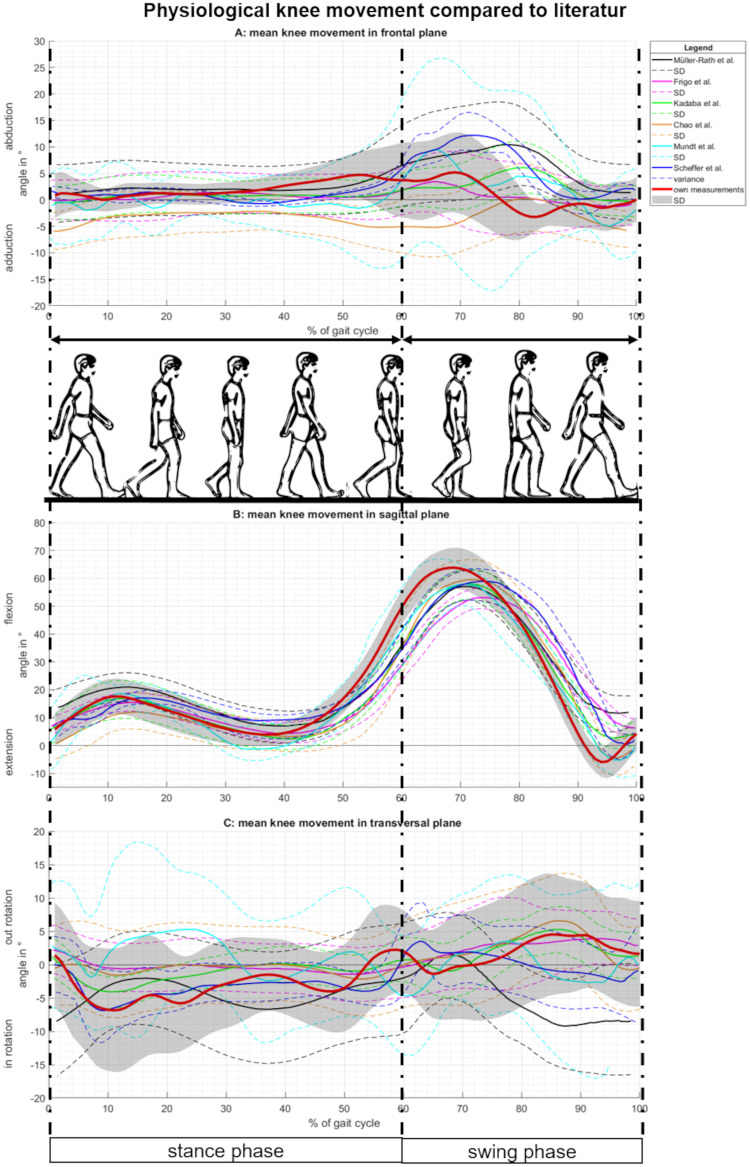
Physiological knee movement (3D) over one gait cycle measured for different publications. (A) Knee ab-/ adduction of the knee during movement in the frontal plane. (B) Knee flexion/ extension of the knee during movement in the sagittal plane. (C) Knee inner/ outer rotation of the knee during movement in the transversal plane.

To evaluate our test setup and results, our data involving the right knee movement in the frontal plane during walking was compared with previous data from literature [23,30–34] (Fig 3).

As the results of our test setup showed a strong correlation to existing data from previous literature, we concluded that using an IMU-system, a valid detection of the brace’s influence on the knee during walking was possible.

### Changes caused by the brace

**Fig 4:**
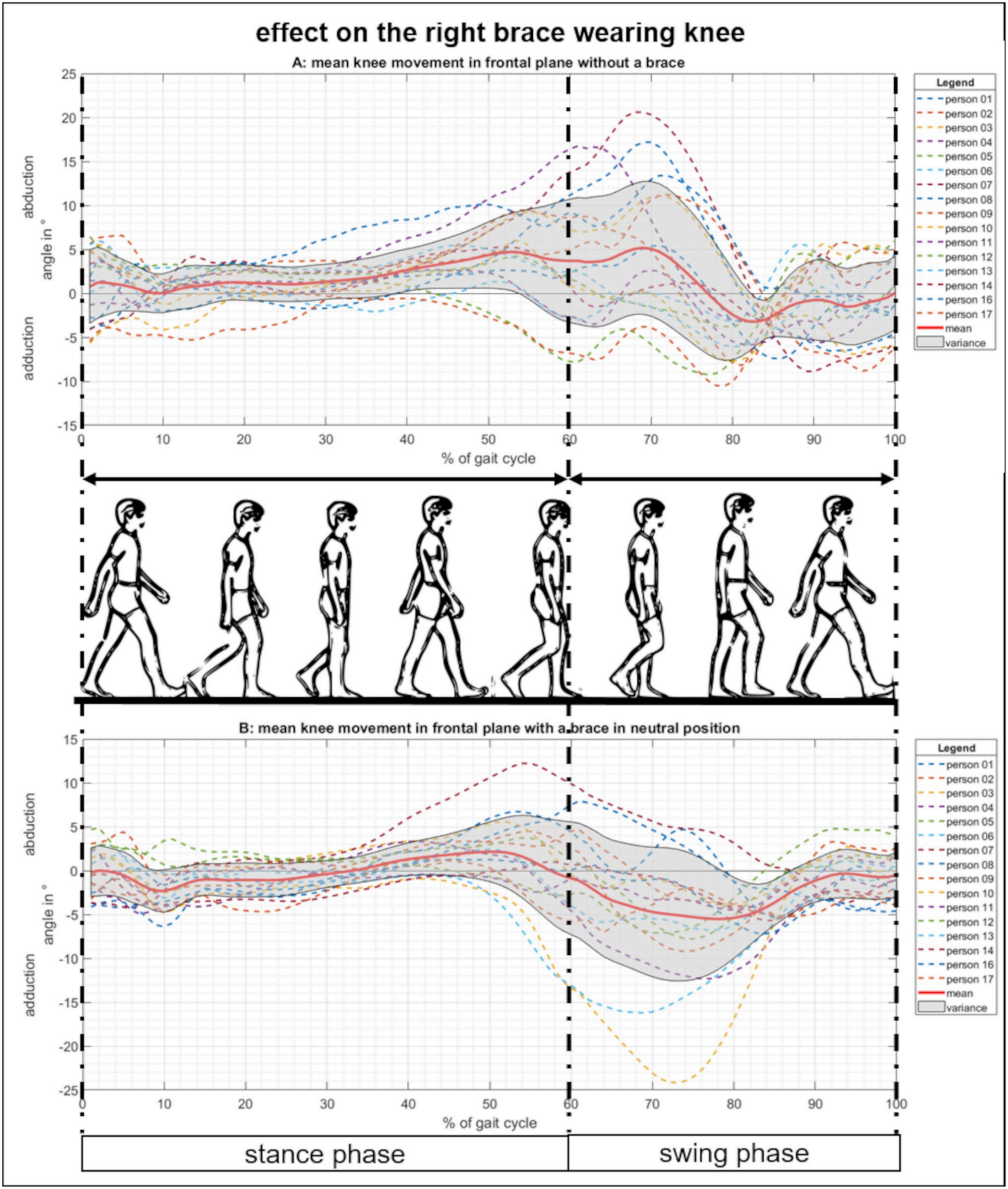
Movement of the knee in the frontal plane with and without a brace. (A) Knee ab-/adduction over one gait cycle for physiological walking without a brace. (B) Knee ab-/adduction while wearing the knee brace in a neutral position.

**Fig 5:**
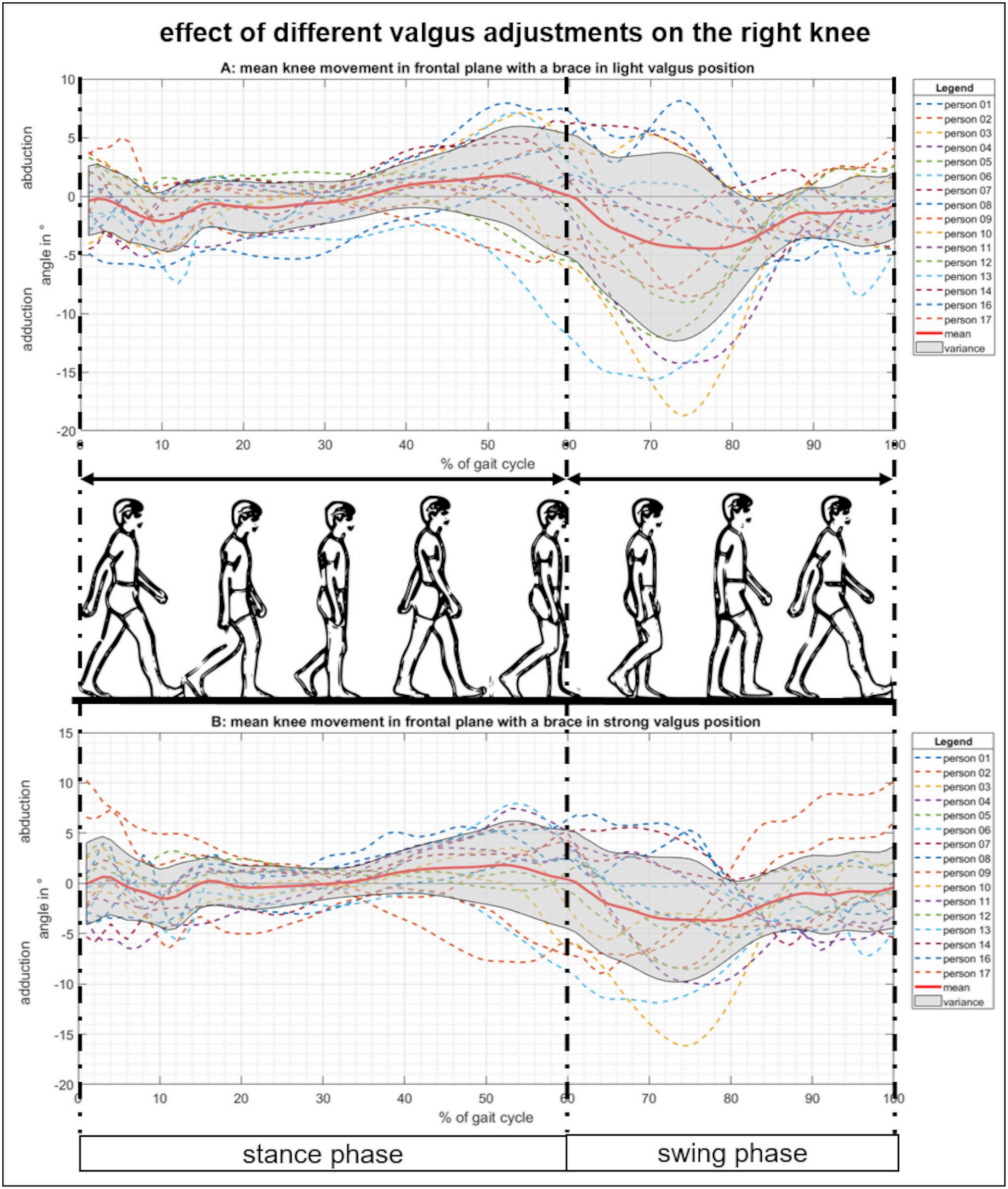
Movement of the right knee in the frontal plane with a valgus adjusted brace. (A) Knee ab-/adduction over one gait cycle with the brace in a light valgus adjustment. (B) Knee ab-/adduction with the brace in a strong valgus adjustment.

**Fig 6:**
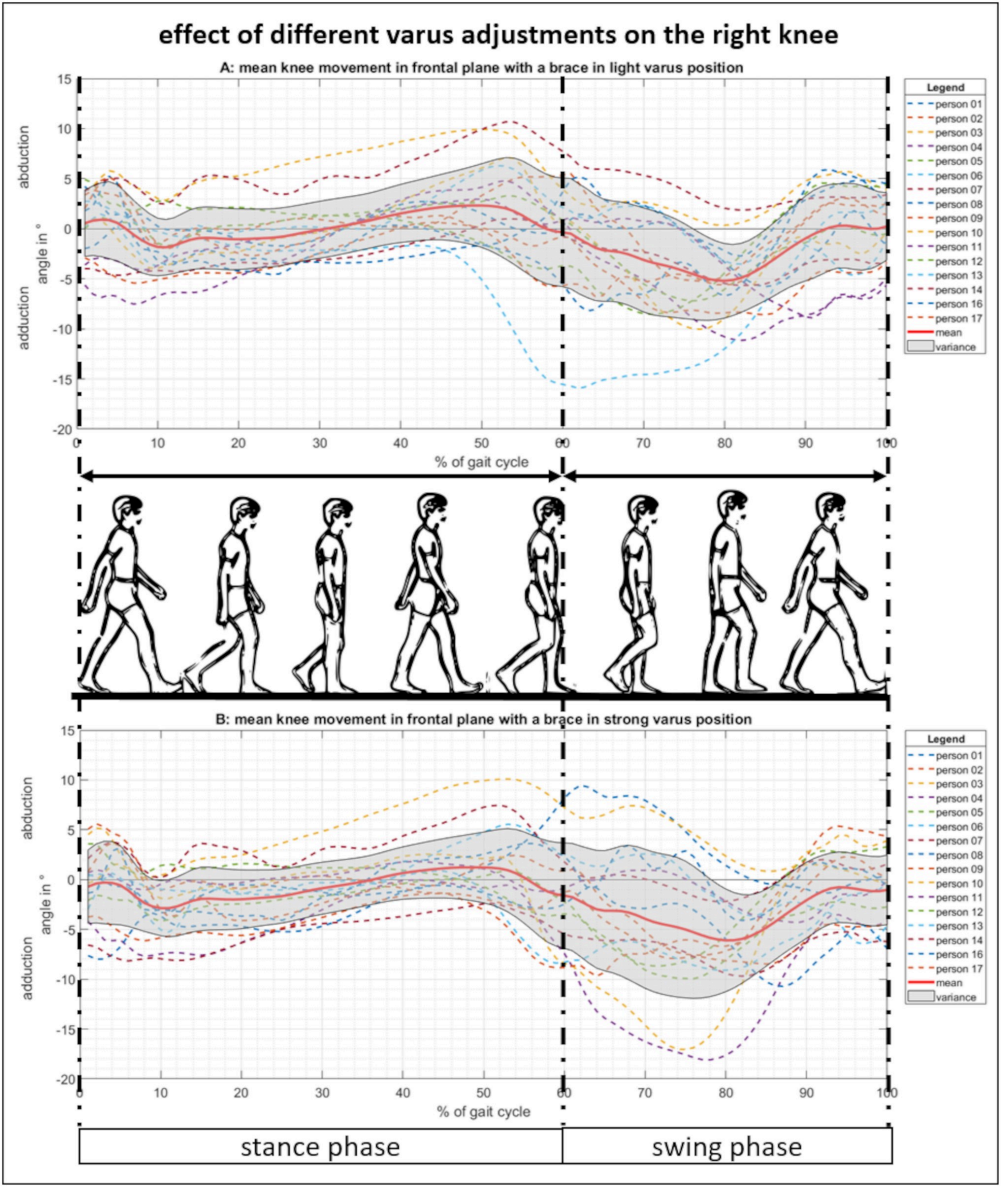
Movement of the right knee in the frontal plane with a varus adjusted brace. (A) Knee ab-/adduction over one gait cycle with the brace in a light varus adjustment. (B) Knee ab-/adduction with the brace in a strong varus adjustment.

In the first step of influence testing, to see the pure bracing effects, the movement of the right knee in a neutral position with and without the brace was compared (Fig 4). The descriptive statistics can be found in S1 Appendix. Descriptive Statistics. From the data, we identified one participant (p15) as an outlier, and therefore, excluded them from further analysis. (Analysis including participant p15 was presented in S2 Appendix. Data with the outlier. Including descriptive statistics and figures compared to Figs 4–6). To compare between measurement conditions, mean values derived from all participant data were calculated and presented with its variance, or mean plus and minus one standard deviation.

The statistical pairwise comparison (Wilcoxon test) showed that the abduction (maximum distance moved in the frontal plane away from the body’s midline) of the right knee was significantly reduced by the brace (Table 1). In contrast, adduction (minimum distance moved in the frontal plane towards the midline of the body) and the range of motion (ROM) (full movement potential in the frontal plane between maximum abduction and adduction) showed a not significant medium effect with slightly rising maximum adduction and ROM. Further, we measured a significantly strong effect of the brace on the ROM in the sagittal and transversal plane: the maximum knee flexion was reduced and the outer rotation was significantly reduced (Table 1).

**Table 1:** Statistic results of the Wilcoxon test used to compare between the measurements taken without a brace and the brace in a neutral position.

Parameter		N	Median in °		z	p-value/ significance (2-tailed)	Effect size r² in %
			Without	With			
**Frontal plane**	Max / abduction	16	6.24	3.52	-2.9	0.004	53
	Min / adduction	16	-6.73	-6.89	-0.78	0.438	4
	ROM	16	15.79	11.75	-1.45	0.148	13
**Sagittal plane**	Max / flexion	16	64.15	62.93	-1.91	0.056	23
	Min / extension	16	-7.38	-4.68	-1.81	0.070	20
	ROM	16	72.61	66.23	-3.05	0.002	58
**Trans-verse plane**	Max / out rotation	16	12.10	5.18	-2.33	0.020	34
	Min / in rotation	16	-8.29	-7.34	-1.09	0.278	7
	ROM	16	18.70	13.61	-2.95	0.003	54

### Effect of the different brace adjustments

In order to analyze the effects of different brace adjustments in the frontal plane, the brace was tuned to light and strong valgus (Fig 5) or varus settings (Fig 6) while the motion of the right knee over one gait cycle was tracked.

By comparing the measurements obtained with the brace in neutral, light, and strong adjustments for valgus or varus pressure relief, we were able to analyze the effect of different brace adjustments on the gait cycle. When the brace was in a valgus setting, a significant effect on the maximum knee flexion, outward knee rotation, and ROM in the transverse plane was observed (Table 2). A varus adjustment of the brace led to significant changes in the maximum abduction, flexion, extension, and inward rotation of the knee (Table 2). A significant reduction of abduction and inward rotation was observed between light and strong varus settings (Table 2). Compared to a neutrally adjusted brace, light and strong varus adjustments significantly reduced maximum knee flexion while extension was significantly increased with a light varus adjustment (Table 2).

**Table 2:** Statistic results of the Friedman-Test to analyze the effect of different brace adjustments.

Brace adjustment	parameter		N	Chi-squared	Sig (2-tailed)	Post hoc pairwise comparison: Sig		
						neutral vs light	neutral vs strong	strong vs light
**valgus**	Frontal plane	Max / abduction	16	1.625	0.444	./.	./.	./.
		Min / adduction	16	0.375	0.829	./.	./.	./.
		ROM	16	0.000	1.000	./.	./.	./.
	Sagittal plane	Max / flexion	16	8.000	0.018	0.472	0.014	0.472
		Min / extension	16	5.375	0.068	./.	./.	./.
		ROM	16	2.375	0.305	./.	./.	./.
	Trans-verse plane	Max / out rotation	16	11.375	0.003	0.472	0.002	0.155
		Min / in rotation	16	2.000	0.368	./.	./.	./.
		ROM	16	6.500	0.039	1.000	0.040	0.231
**varus**	Frontal plane	Max / abduction	16	6.125	0.047	0.648	0.648	0.040
		Min / adduction	16	4.875	0.087	./.	./.	./.
		ROM	16	0.875	0.646	./.	./.	./.
	Sagittal plane	Max / flexion	16	9.500	0.009	0.014	0.040	1.000
		Min / extension	16	10.500	0.005	0.004	0.102	0.867
		ROM	16	3.875	0.144	./.	./.	./.
	Trans-verse plane	Max / out rotation	16	4.875	0.087	./.	./.	./.
		Min / in rotation	16	6.500	0.039	0.231	1.000	0.040
		ROM	16	0.375	0.829	./.	./.	./.

A valgus adjustment for the brace provides pressure relief by shifting load to the lateral condyles by increasing abduction and/or decreasing adduction. For a varus brace, the effect is reversed by load shifting to the medial condyles through increased adduction and/or decreased abduction. Adjusting the brace to different settings affects the kinematics of the knee, and in Table 3, we presented the number of cases that exhibited the desired kinematic changes caused by the different brace manipulations. In 56% of cases of valgus and 38% of varus adjustments, we found the desired effects presented themselves with both strong and light brace settings (Table 3).

**Table 3:** Percent of cases (number of attendees) showing the desired effect caused by brace manipulation.

	Light Valgus	Strong Valgus	Light Varus	Strong Varus	Both Valgus	Both Varus
abduction	44% (7)	50% (8)	38% (6)	63% (10)	31% (5)	31% (5)
adduction	56% (9)	50% (8)	25% (4)	44% (7)	38% (6)	13% (2)
any effect	69% (11)	75% (12)	50% (8)	69% (11)	56% (9)	38% (6)

The first row presents the percent of cases where the maximum abduction is increased by a valgus adjustment and reduced by a varus adjustment. The second row lists the percent of cases where the opposite effects were observed for maximum adduction. The third row is the percent of all cases where any effect was measured. All cases are compared to measurements taken with the brace in a neutral setting.

### Individual movement pattern

**Fig 7:**
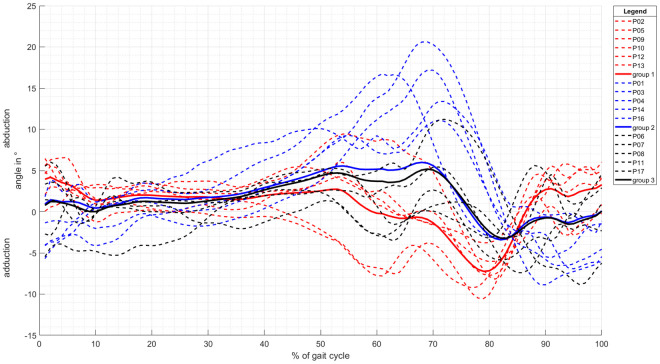
Grouped knee movement in the frontal plane for physiological walking.

Analyzing the movement pattern of the knee in the frontal plane visually, we found individual patterns. Based on the curve progression of the ab-/ adduction angle, we were able to identify three different groups. The subjective classification is shown in Fig 7 and mainly based on the peak count, height, prominence, and orientation. When analyzing the mean curve progressions, it can be seen that the difference between groups 2 and 3 is small, but group 1 shows an entirely different movement pattern.

## Discussion

Using a knee brace to support the mobility of OA patients is a common functional and clinical solution. This study provided a comprehensive description of the knee joint motion in a three-dimensional field while level walking without and with wearing a knee brace in different settings. Our hypothesis supports the observation of decreased knee flexion and adapted knee movement patterns in the frontal plane caused by varus or valgus pressure relief brought forth by the brace.

To show the effectiveness of our measurement setup, we compared our reference data, measurements from healthy participants walking without a brace, to previously published data (Fig 3). The angle progressions of the knee movement in all three dimensions are comparable. Different measurement methods and techniques are responsible for small differences in the angle progression curves. In the frontal and transverse plane, there are only small movements with a high variance. Therefore, measurement errors attributed to IMUs, like soft tissue artifacts [35] or magnetic disturbance [23], have a higher influence on the data. When analyzing the knee flexion/ extension in the sagittal plane, a small shift of the main peak towards the stance phase was observed which could be the result of our toe-off detection. As described, difficulties based on kinematic data arise when detecting gait events like initial or terminal contact [28,29]. Nevertheless, except for a small shift, our measurement data is comparable with previously published data, which supports the validity of our measurement method and results. Therefore, it can be concluded that IMUs have the potential to provide a low-cost, easy-to-use, and portable device for movement analysis.

Our results have shown us that wearing a knee brace significantly reduces the maximum knee abduction while increasing the maximum adduction. Consequently, the ROM of the knee in the frontal plane is reduced. Since this is the overall effect of the knee brace, the different effects with respect to either a varus or valgus manipulation can be explained (Tables 2 and 3). A valgus manipulation, similar to the neutral setting, of the knee brace produced higher abduction and lower adduction. In contrast, a varus manipulation produced higher adduction and lower abduction. The brace itself supports a valgus orientation of the knee, thus, varus pressure relief only leads to the proposed frontal plane movement in 38% of cases. For valgus adjusted braces, 56% produced the desired movement changes. Brouwer et al. also described the difference between varus and valgus bracing, concluding that valgus bracing was more effective than varus bracing [36]. Toda et. al suggested a correlation between joint realignment and clinical improvement [37]. However, it was noted that most previous studies concentrated on the clinical effects of valgus bracing in patients with medial knee OA [6,14–17,19,38].

By analyzing the effects of the knee brace on the complete knee movement in three dimensions, a significant reduction of the maximum abduction and in the ROM through the sagittal and transverse plane was discovered (Table 2). These reductions are considered as movement restrictions, which can be uncomfortable for the patient, leading to poor patient compliance described in a previous publication [19].

We analyzed the frontal knee movement in detail in an attempt to explain the different effects of the brace. As described before, the mean angle progression of the knee ab-/ adduction is comparable to previously published data (Fig 3). However, individual movement patterns of the knee ab-/ adduction show some differences between the participants (Fig 7). In previous studies, only the mean angle progression and/ or the angel curve of one exemplary subject was presented. Different angle progressions for different participants were not described or discussed. However, these individual movement patterns can be the cause of various effects on the brace and the different brace manipulations. Since each patient displays different movement patterns of the knee in the frontal plane, they also react differently to therapy. Therefore, individualized therapy becomes more important. IMUs, as used in this study, are an option for individual, or at least personalized, therapy monitoring.

Our approach includes some inherent limitations. In contrast to previous studies, we decided to analyze healthy participants instead of ‘real’ patients. Therefore, we were able to analyze the effect of the brace and different brace adjustments on the knee joint itself, without confounding OA effects. However, we were not able to analyze the effectiveness of bracing on patients with OA or any accompanying long term effects. Since this was only a pilot study to analyze the bracing effect on healthy knee joints, the sample size was small with a majority of men. The limitations of our measurement setup using portable IMUs have already been discussed.

## Conclusion

As the number of patients with knee OA increases, finding an effective and low-cost treatment becomes more and more critical. Our results advocate the functionality and effectiveness of a knee brace for varus/ valgus pressure relief. Moreover, the measurement setup is shown to be a low-cost, easy-to-use option for detailed analysis of the individual effects of assistive devices. Such a solution is important, especially since our results show the individual effects of wearing a knee brace, like unique movement patterns and compensation strategies.

## Supporting information

S1 Appendix Descriptive statistics. (DOCX)

S2 Appendix Data including the outlier. (DOCX)
