# Supplementary material for: The effect of a knee brace in dynamic motion—An instrumented gait analysis
Source: PLoS One. 2020 Sep 10;15(9):e0238722. doi: 10.1371/journal.pone.0238722 (PMC7482934; doi:10.1371/journal.pone.0238722)
Supplement: S1 Appendix — (DOCX) [file pone.0238722.s001.docx]

Table 1: Descriptive statistics of the knee movement (extrema) of all included participants.

|  | Brace | N | Minimum | Maximum | Mean | Std.-Deviation | Shapiro-Wilk sigma |
| --- | --- | --- | --- | --- | --- | --- | --- |
| maximal knee angle in frontal plane | without | 16 | 2,68 | 20,61 | 9,19 | 5,33 | 0,035 |
|  | neutral orientation | 16 | 0,99 | 12,21 | 4,20 | 2,92 | 0,038 |
|  | light valgus | 16 | -1,36 | 8,14 | 3,94 | 2,63 | 0,783 |
|  | strong valgus | 16 | 0,27 | 10,21 | 4,78 | 2,81 | 0,826 |
|  | light varus | 16 | 0,62 | 10,67 | 4,93 | 2,79 | 0,453 |
|  | strong varus | 16 | -2,39 | 10,08 | 3,36 | 3,71 | 0,706 |
| minimal knee angle in frontal plane | without | 16 | -10,53 | -2,50 | -6,52 | 2,41 | 0,586 |
|  | neutral orientation | 16 | -24,17 | -3,67 | -8,23 | 5,35 | 0,001 |
|  | light valgus | 16 | -18,71 | -2,26 | -8,13 | 4,78 | 0,103 |
|  | strong valgus | 16 | -16,16 | -2,14 | -7,44 | 3,36 | 0,285  285 |
|  | light varus | 16 | -15,94 | 1,86 | -6,75 | 4,26 | 0,701 |
|  | strong varus | 16 | -18,09 | -0,36 | -8,12 | 4,81 | 0,314 |
| maximal knee angle in sagittal plane | without | 16 | 52,26 | 73,43 | 63,98 | 7,20 | 0,154 |
|  | neutral orientation | 16 | 49,61 | 70,75 | 61,05 | 6,45 | 0,350 |
|  | light valgus | 16 | 47,79 | 69,93 | 58,96 | 6,87 | 0,444 |
|  | strong valgus | 16 | 45,59 | 66,69 | 57,95 | 6,77 | 0,329 |
|  | light varus | 16 | 45,18 | 64,96 | 56,01 | 7,40 | 0,016 |
|  | strong varus | 16 | 36,70 | 71,11 | 55,01 | 8,56 | 0,800 |
| minimal knee angle in sagittal plane | without | 16 | -13,28 | 0,95 | -6,94 | 4,70 | 0,304 |
|  | neutral orientation | 16 | -13,54 | 3,51 | -4,86 | 4,85 | 0,953 |
|  | light valgus | 16 | -14,14 | 3,44 | -6,60 | 4,50 | 0,774 |
|  | strong valgus | 16 | -16,50 | 3,89 | -7,21 | 5,00 | 0,917 |
|  | light varus | 16 | -18,13 | 2,14 | -7,97 | 5,56 | 0,572 |
|  | strong varus | 16 | -15,91 | 2,75 | -8,34 | 5,22 | 0,766 |
| maximal knee angle in transversal plane | without | 16 | -4,76 | 19,32 | 9,23 | 7,42 | 0,295 |
|  | neutral orientation | 16 | -6,91 | 13,50 | 5,24 | 4,98 | 0,255 |
|  | light valgus | 16 | 0,03 | 16,36 | 7,05 | 5,13 | 0,468 |
|  | strong valgus | 16 | 1,94 | 17,29 | 9,57 | 4,86 | 0,403 |
|  | light varus | 16 | -5,99 | 11,82 | 3,74 | 4,83 | 0,936 |
|  | strong varus | 16 | -1,41 | 14,61 | 5,41 | 5,07 | 0,258 |
| minimal knee angle in transversal plane | without | 16 | -24,17 | 2,36 | -10,23 | 7,69 | 0,687 |
|  | neutral orientation | 16 | -19,71 | -0,72 | -8,61 | 6,08 | 0,280 |
|  | light valgus | 16 | -12,45 | 0,50 | -7,12 | 3,85 | 0,619 |
|  | strong valgus | 16 | -14,76 | 0,19 | -6,03 | 4,69 | 0,215 |
|  | light varus | 16 | -25,65 | -0,66 | -9,65 | 6,59 | 0,208 |
|  | strong varus | 16 | -22,68 | 2,23 | -8,31 | 5,80 | 0,731 |

Table 2: descriptive statistics of the knee movement (ROM) of all included participants

|  | Brace | N | Minimum | Maximum | Mean | Std.-Deviation | Shapiro-Wilk sigma |
| --- | --- | --- | --- | --- | --- | --- | --- |
| knee ROM in frontal plane | without | 16 | 8,01 | 29,48 | 15,71 | 6,12 | 0,427 |
|  | neutral orientation | 16 | 6,69 | 26,93 | 12,43 | 5,06 | 0,025 |
|  | light valgus | 16 | 5,16 | 21,65 | 12,08 | 4,16 | 0,754 |
|  | strong valgus | 16 | 7,86 | 19,77 | 12,21 | 3,87 | 0,069 |
|  | light varus | 16 | 6,64 | 18,69 | 11,68 | 3,39 | 0,339 |
|  | strong varus | 16 | 6,10 | 22,22 | 11,47 | 4,11 | 0,065 |
| knee ROM in sagittal plane | without | 16 | 61,39 | 78,53 | 70,92 | 5,69 | 0,110 |
|  | neutral orientation | 16 | 55,01 | 75,42 | 65,91 | 5,83 | 0,758 |
|  | light valgus | 16 | 55,51 | 75,59 | 65,56 | 5,04 | 0,801 |
|  | strong valgus | 16 | 54,52 | 74,56 | 65,16 | 5,31 | 0,332 |
|  | light varus | 16 | 53,13 | 74,26 | 63,98 | 6,69 | 0,616 |
|  | strong varus | 16 | 49,66 | 76,50 | 63,35 | 7,27 | 0,852 |
| knee ROM in transversal plane | without | 16 | 12,87 | 28,90 | 19,46 | 4,65 | 0,770 |
|  | neutral orientation | 16 | 8,37 | 19,87 | 13,85 | 4,07 | 0,143 |
|  | light valgus | 16 | 8,89 | 19,22 | 14,17 | 3,78 | 0,073 |
|  | strong valgus | 16 | 9,45 | 28,20 | 15,61 | 4,92 | 0,121 |
|  | light varus | 16 | 7,94 | 21,41 | 13,39 | 4,41 | 0,117 |
|  | strong varus | 16 | 6,87 | 23,00 | 13,71 | 4,85 | 0,070 |
