# Supplementary material for: The effect of a knee brace in dynamic motion—An instrumented gait analysis
Source: PLoS One. 2020 Sep 10;15(9):e0238722. doi: 10.1371/journal.pone.0238722 (PMC7482934; doi:10.1371/journal.pone.0238722)
Supplement: S2 Appendix — (DOCX) [file pone.0238722.s002.docx]

Table 1: Descriptive statistics of the knee movement (extrema) in frontal plane of all participants.

|  | Brace | N | Minimum | Maximum | Mean | Std.-Deviation |
| --- | --- | --- | --- | --- | --- | --- |
| maximal knee angle in frontal plane | without | 17 | 2,68 | 30,03 | 10,41 | 7,23 |
|  | neutral orientation | 17 | 0,99 | 27,65 | 5,58 | 6,35 |
|  | light valgus | 17 | -1,36 | 26,37 | 5,26 | 6,01 |
|  | strong valgus | 17 | 0,27 | 27,41 | 6,11 | 6,12 |
|  | light varus | 17 | 0,62 | 18,84 | 5,75 | 4,32 |
|  | strong varus | 17 | -2,39 | 26,35 | 4,71 | 6,64 |
| minimal knee angle in frontal plane | without | 17 | -10,53 | -2,50 | -6,38 | 2,41 |
|  | neutral orientation | 17 | -24,17 | -3,67 | -8,03 | 5,24 |
|  | light valgus | 17 | -18,71 | -2,26 | -7,96 | 4,68 |
|  | strong valgus | 17 | -16,16 | -2,14 | -7,45 | 3,25 |
|  | light varus | 17 | -15,94 | 1,86 | -6,87 | 4,15 |
|  | strong varus | 17 | -18,09 | -0,36 | -8,13 | 4,66 |
| maximal knee angle in sagittal plane | without | 17 | 52,26 | 73,43 | 63,81 | 7,01 |
|  | neutral orientation | 17 | 49,61 | 70,75 | 60,75 | 6,37 |
|  | light valgus | 17 | 47,79 | 69,93 | 58,83 | 6,67 |
|  | strong valgus | 17 | 45,59 | 66,69 | 57,93 | 6,55 |
|  | light varus | 17 | 45,18 | 64,96 | 56,00 | 7,16 |
|  | strong varus | 17 | 36,70 | 71,11 | 54,87 | 8,31 |
| minimal knee angle in sagittal plane | without | 17 | -13,28 | 0,95 | -6,83 | 4,57 |
|  | neutral orientation | 17 | -13,54 | 3,51 | -4,70 | 4,74 |
|  | light valgus | 17 | -14,14 | 3,44 | -6,38 | 4,45 |
|  | strong valgus | 17 | -16,50 | 3,89 | -6,93 | 4,98 |
|  | light varus | 17 | -18,13 | 2,14 | -7,69 | 5,50 |
|  | strong varus | 17 | -15,91 | 2,75 | -7,88 | 5,40 |
| maximal knee angle in transversal plane | without | 17 | -4,76 | 19,32 | 8,84 | 7,37 |
|  | neutral orientation | 17 | -6,91 | 13,50 | 4,81 | 5,14 |
|  | light valgus | 17 | 0,03 | 16,36 | 7,38 | 5,14 |
|  | strong valgus | 17 | 1,94 | 17,29 | 9,85 | 4,85 |
|  | light varus | 17 | -5,99 | 11,82 | 3,64 | 4,70 |
|  | strong varus | 17 | -1,75 | 14,61 | 4,99 | 5,20 |
| minimal knee angle in transversal plane | without | 17 | -24,17 | 2,36 | -10,42 | 7,49 |
|  | neutral orientation | 17 | -20,79 | -0,72 | -9,33 | 6,59 |
|  | light valgus | 17 | -15,55 | 0,50 | -7,61 | 4,25 |
|  | strong valgus | 17 | -14,76 | 0,19 | -6,44 | 4,84 |
|  | light varus | 17 | -25,65 | -0,66 | -9,90 | 6,46 |
|  | strong varus | 17 | -22,68 | 2,23 | -8,78 | 5,95 |

Table 2: descriptive statistics of the knee movement (ROM) of all included participants

|  | Brace | N | Minimum | Maximum | Mean | Std.-Deviation |
| --- | --- | --- | --- | --- | --- | --- |
| knee ROM in frontal plane | without | 17 | 8,01 | 34,12 | 16,79 | 7,42 |
|  | neutral orientation | 17 | 6,69 | 32,59 | 13,61 | 6,92 |
|  | light valgus | 17 | 5,16 | 31,61 | 13,23 | 6,22 |
|  | strong valgus | 17 | 7,86 | 35,07 | 13,56 | 6,69 |
|  | light varus | 17 | 6,64 | 27,51 | 12,61 | 5,05 |
|  | strong varus | 17 | 6,10 | 34,61 | 12,83 | 6,88 |
| knee ROM in sagittal plane | without | 17 | 61,39 | 78,53 | 70,64 | 5,63 |
|  | neutral orientation | 17 | 55,01 | 75,42 | 65,45 | 5,96 |
|  | light valgus | 17 | 55,51 | 75,59 | 65,21 | 5,09 |
|  | strong valgus | 17 | 54,52 | 74,56 | 64,86 | 5,28 |
|  | light varus | 17 | 53,13 | 74,26 | 63,69 | 6,58 |
|  | strong varus | 17 | 49,66 | 76,50 | 62,75 | 7,46 |
| knee ROM in transversal plane | without | 17 | 12,87 | 28,90 | 19,26 | 4,58 |
|  | neutral orientation | 17 | 8,37 | 19,87 | 14,13 | 4,11 |
|  | light valgus | 17 | 8,89 | 28,11 | 14,99 | 4,98 |
|  | strong valgus | 17 | 9,45 | 28,20 | 16,29 | 5,54 |
|  | light varus | 17 | 7,94 | 21,41 | 13,53 | 4,31 |
|  | strong varus | 17 | 6,87 | 23,00 | 13,77 | 4,70 |


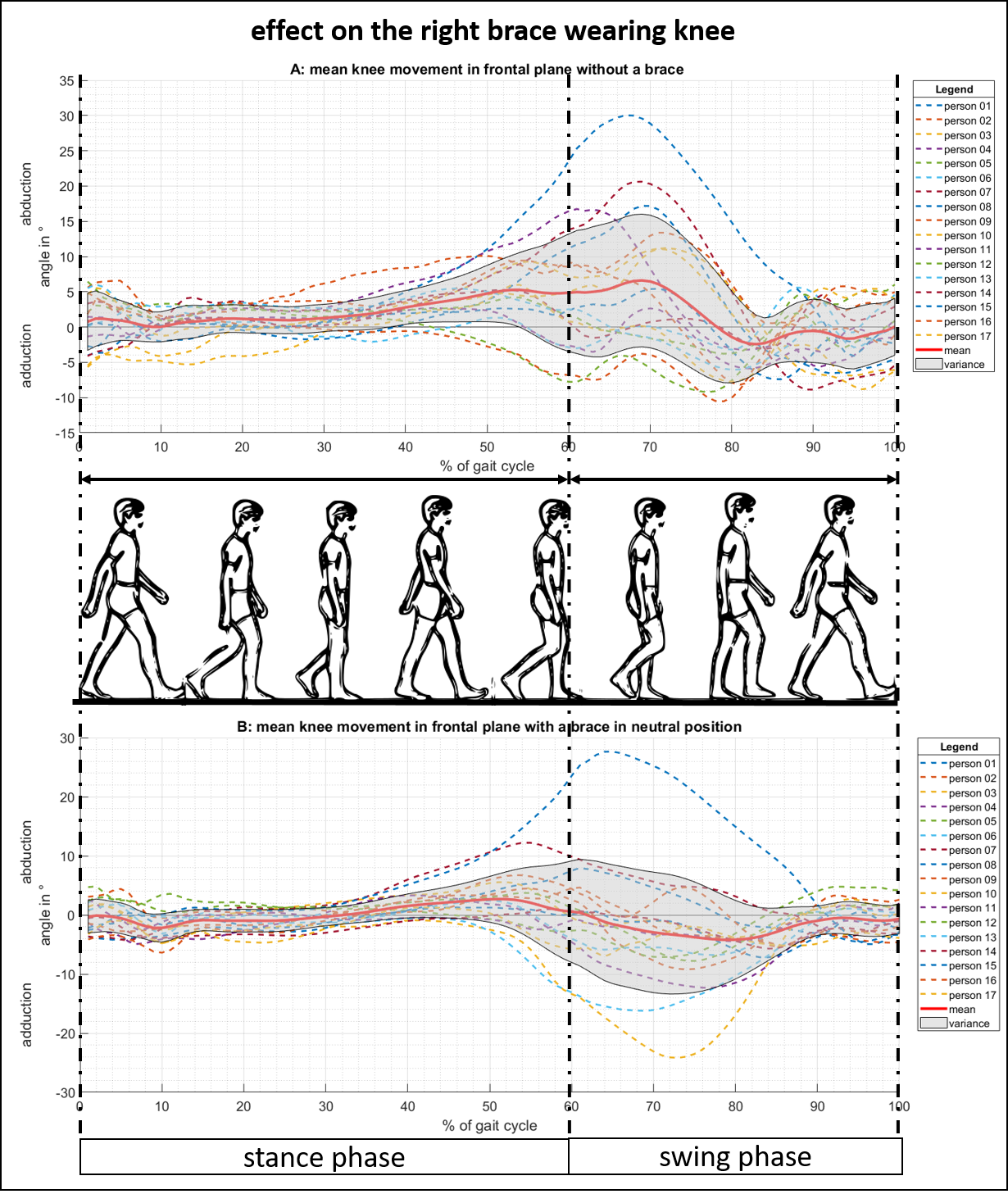


Fig 1. Frontal plane knee movement of the right knee with and without a brace.

*(A) The knee ab-/adduction over one gait cycle in the case of physiological walking without a brace. (B) The knee ab-/adduction in the case of wearing the knee brace in neutral position.*


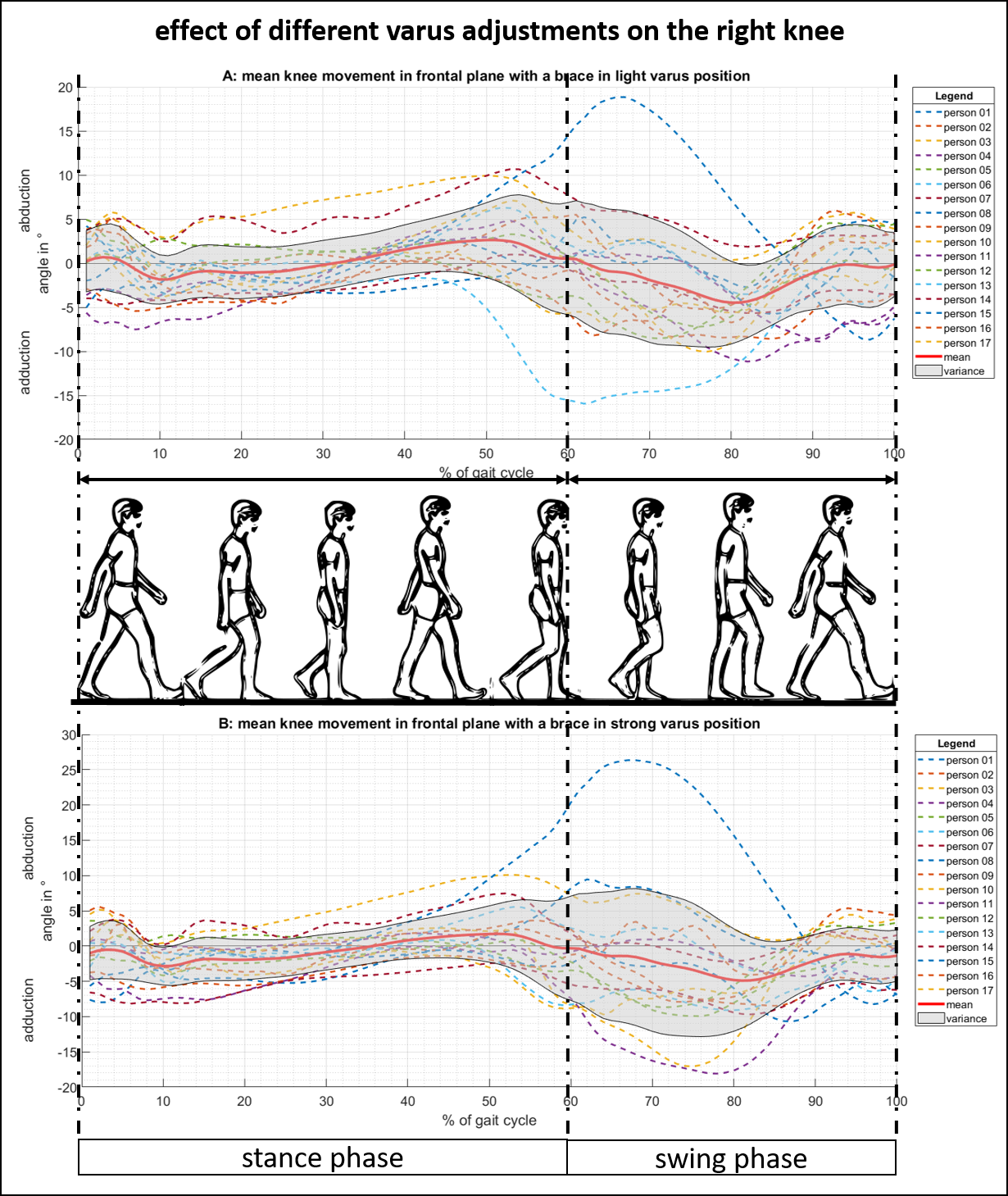


Fig 2. Frontal plane knee movement of the right knee with a valgus adjusted brace.

*(A) The knee ab-/adduction over one gait cycle in the case of a brace in a light valgus adjustment. (B) The knee ab-/adduction in the case of a brace in a strong valgus adjustment.*

**
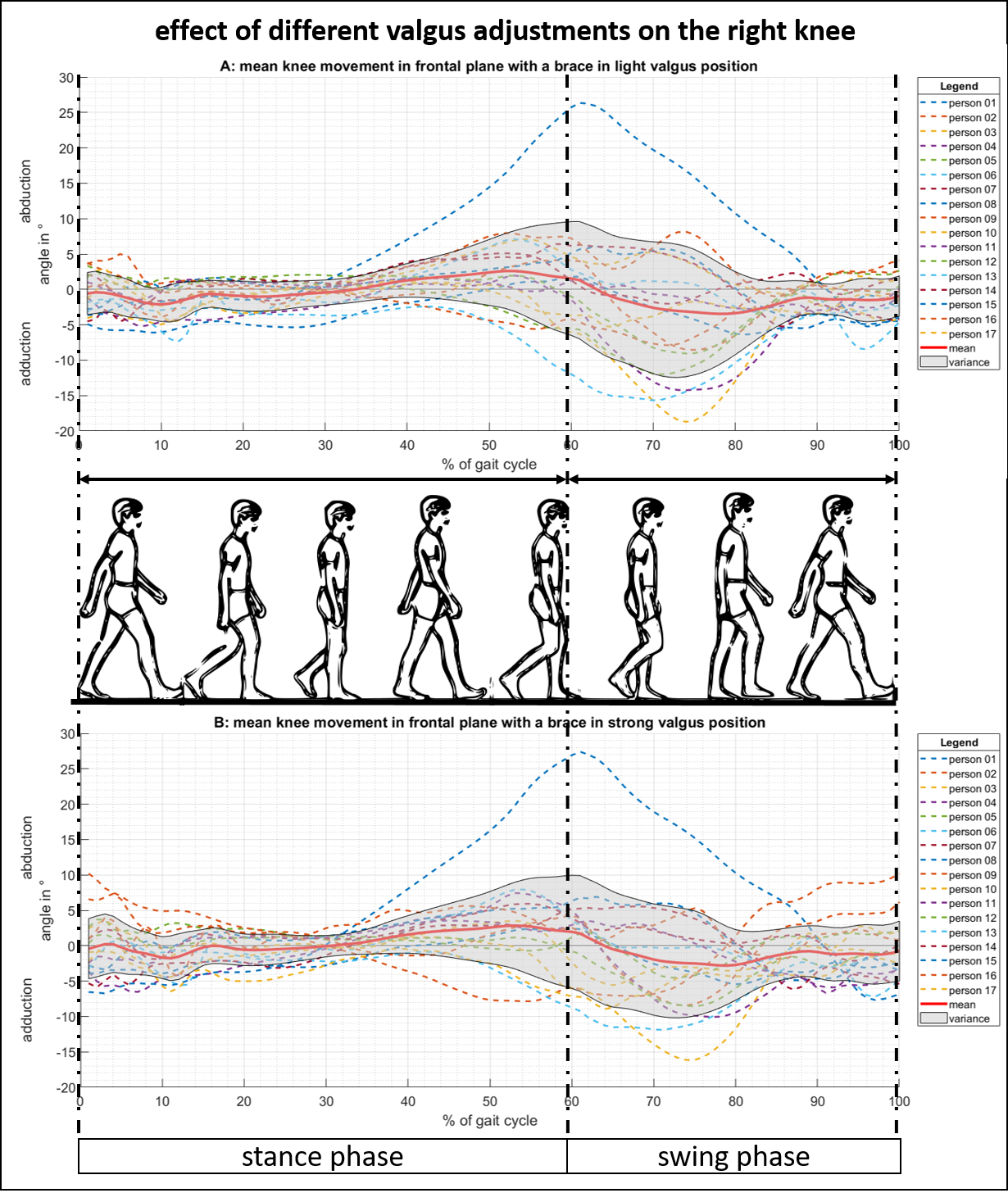
**

Fig 3. Frontal plane knee movement of the right knee with a valgus adjusted brace.

*(A) The knee ab-/adduction over one gait cycle in the case of a brace in a light varus adjustment. (B) The knee ab-/adduction in the case of a brace in a strong varus adjustment.*
